# Circulating Nucleosomes and Nucleosome Modifications as Biomarkers in Cancer

**DOI:** 10.3390/cancers9010005

**Published:** 2017-01-08

**Authors:** Peter McAnena, James A. L. Brown, Michael J. Kerin

**Affiliations:** Discipline of Surgery, Lambe Institute for Translational Research, School of Medicine, National University of Ireland Galway, H91 YR71 Galway, Ireland; p.mcanena1@nuigalway.ie (P.M.); michael.kerin@nuigalway.ie (M.J.K.)

**Keywords:** breast cancer, histone, colorectal, circulating, biomarker, miRNA, microRNA, posttranslational, modification

## Abstract

Traditionally the stratification of many cancers involves combining tumour and clinicopathological features (e.g., patient age; tumour size, grade, receptor status and location) to inform treatment options and predict recurrence risk and survival. However, current biomarkers often require invasive excision of the tumour for profiling, do not allow monitoring of the response to treatment and stratify patients into broad heterogeneous groups leading to inconsistent treatment responses. Here we explore and describe the benefits of using circulating biomarkers (nucleosomes and/or modifications to nucleosomes) as a non-invasive method for detecting cancer and monitoring response to treatment. Nucleosomes (DNA wound around eight core histone proteins) are responsible for compacting our genome and their composition and post-translational modifications are responsible for regulating gene expression. Here, we focus on breast and colorectal cancer as examples where utilizing circulating nucleosomes as biomarkers hold real potential as liquid biopsies. Utilizing circulating nucleosomes as biomarkers is an exciting new area of research that promises to allow both the early detection of cancer and monitoring of treatment response. Nucleosome-based biomarkers combine with current biomarkers, increasing both specificity and sensitivity of current tests and have the potential to provide individualised precision-medicine based treatments for patients.

## 1. Introduction

Cancer leads to the death of around 8.2 million people annually world-wide [1], despite advances in detection and treatment. Cancer is a group of disorders resulting from aberrant genetic and epigenetic alterations that lead to genomic instability and ultimately uncontrolled cellular proliferation. Chromosomal instability is a major form of genomic volatility and contributes to abnormal chromosomal structure and numbers. Micro-satellite instability and increased frequency of base-pair mutations are other described forms of genomic instability, and these enable the acquisition of the hallmarks of cancer [2]. Epigenetic changes are heritable, functionally relevant changes altering gene activity, without altering the underlying DNA sequence. Epigenetic regulation results from changing the accessibility of DNA and altering chromatin structure through posttranslational modifications of either DNA, or DNA bound proteins such as histones. The most frequently studied epigenetic changes are DNA methylation and posttranslational histone modifications.

The National Cancer Institute defines a biomarker as “a biological molecule found in blood, other body fluids or tissue that is a sign of a normal or abnormal process or of a condition or disease” [3]. A key clinical goal is the identification of biomarkers that can be used prognostically or diagnostically for early disease detection, to inform optimization of chemo/radiotherapy treatment regimes, potentially identify new therapeutic options or highlight novel therapeutic targets. Here we discuss the post-translational modification of nucleosomes and their use as tumour biomarkers and highlight the potential of using quantification of circulating nucleosomes or histone modifications as biomarkers in cancer.

## 2. The Role of Nucleosomes in Packaging DNA

**Figure 1:**
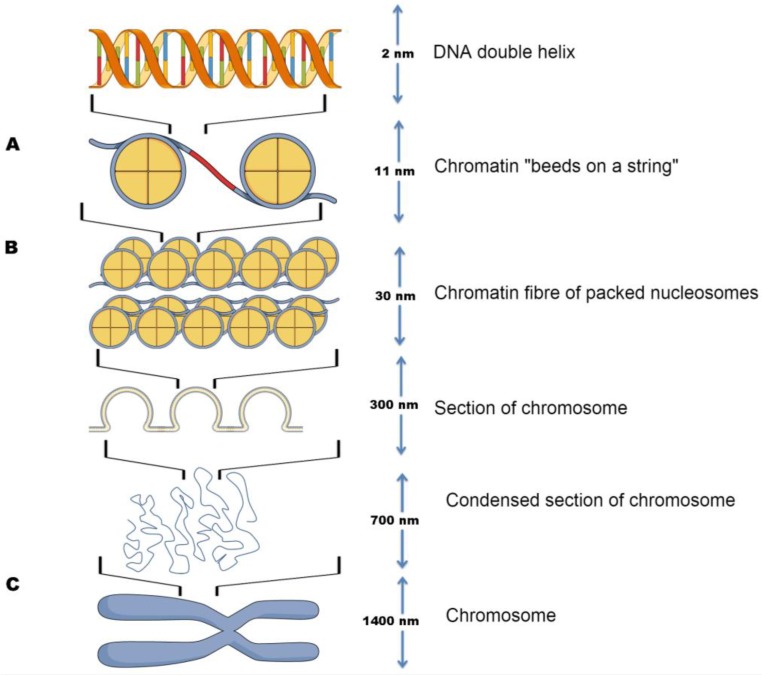
Schematic of DNA compaction levels facilitated by nucleosomes. Left: (**A**) DNA wound around the histone octamer, forming a nucleosome; (**B**) Nucleosomes aggregated into chromatin fibres, which compile into higher order three-dimensional loops and domains; (**C**) Chromatin fibres assembling into chromosomes. Right: indication of the scale of each successive structures compaction.

The first stage of the compaction of genomic DNA in each cell (≈1.8 m) begins by tightly wrapping it around a heterogeneous multi-unit structure, termed a nucleosome. The nucleosome is the core unit of chromatin, first described in 1974 by Kornberg [4]. It consists of an octamer of the four highly conserved core histone proteins (H3, H4, H2A, H2B), joined together by a linker histone H1 with 146 base pairs of DNA wrapped nearly twice around the octamer (Figure 1A). The core histones are predominantly globular, except for a 20–35 amino acid residue “tail” that extend from the surface of the nucleosome, where H2A also contains a ≈37 amino acid carboxy-terminal [5].

The nucleosome functions as the first level of genomic impaction, where a chain of nucleosomes forming chromatin fibres (through the interaction of their tails with adjacent nucleosomes and other proteins) which assemble into higher order three-dimensional nuclear space in a hierarchical manner (forming loops and domains), ultimately assembling chromosomes [6,7,8] (Figure 1B–C).

The nucleosome is a focal point of transcription control and is fundamental to DNA structure and gene regulation [9,10]. Nucleosomes operate as a signalling hub for chromatin-templated processes, acting as a scaffold for chromatin modifying enzymes and individual histones can display a diverse array of post-translational modifications (PTM). These PTM further affect nucleosome stability and regulate protein recruitment, influencing DNA replication and repair [11] and higher order nuclear architecture [12,13,14].

In addition, nucleosomes are a dynamic family, where variation in incorporation of histone variants can mark genomic domains and regulate specific processes [14,15]. Variants of the core histones (H2A, H3 and H1) [16] can be incorporated into nucleosomes in response to specialized requirements or signalling. Nucleosomes in which Centromeric protein A (CENP-A) replaces canonical H3 are the fundamental unit of centromeric chromatin [17], and the H2 variant H2A.Z plays a prominent role in the DNA damage response (DDR) via damaged chromatin reorganization [18,19]. The effects of cellular signalling results in histone PTMs which mediate the recruitment (and action) of non-histone proteins, ultimately regulating the location and chronology of these responses.

## 3. Histone Post-Translational Modifications

Allfrey et al. [20] first postulated that histone modification could contribute to transcription. Importantly, the diversity and complexity of histone PTM and modification sites led to the proposal of the “histone code hypothesis” which proposes that histone PTMs represents a fundamental (in some cases inherited) mechanism regulating chromatin-templated processes, ultimately influencing cell fate and pathological responses [21].

Continued examination of histone PTM mechanisms highlights their central role in all DNA templated processes, producing new insights into the complexity of genomic regulation, maintenance and repair [11]. These PTM include Acetylation (Ac), Methylation (Me), Phosphorylation (P), Ubiquitination (Ub) and Sumoylation (Sumo). Other modifications more recently identified include Glycosylation (Fuc, Gal, GalNAc, Glc, GlcNAc, Man, NeuNAc, Xyl), Homocysteinylation (Hcy) and Crotonylation (Cr) [22]. Many PTM can occur to several degrees e.g., mono-, di- and tri- at a single residue. Over 60 distinct modification sites have been thus far been identified within histones [11]. While acetylation is associated with “open” chromatin and gene transcription, methylation can have a number of functional consequences depending on the residue methylated and the degree of methylation. H3K27me2 and H3K9me3 are associated with gene repression while H3K4me2 and H4K4me3 are associated with active gene expression [8]. To complicate matters further, at so called “bivalent chromatin domains” repressive marks (H3K27) and activating marks (H3K4) can coexist. For a selection of specific PTM sites investigated in cancer, see Table 1 (Histone 2B), Table 2 (Histone 3) and Table 3 (Histone 4).

Currently, three categories of histone modification enzymes are recognized. “Writers” such as histone acetyltransferases which add modifications, “Erasers” such as deacetlylases [23] which remove PTM and “Readers [24]” such as 53BP1 [25] which recognize chromatin bound PTM marks though specific PTM-specific binding domains.

Histone “Writers” disrupt inter-nucleosome and intra-nucleosome contact by causing chromatin to “relax”, becoming more accessible. For example, acetylation acts by causing a reduction in the electrostatic interaction between negatively charged DNA and the lysine residue, leading to more “open” chromatin formation. This provides access to chromatin for process such as transcription or DNA repair. An example of a Writer is Tip60, a histone acetyltransferase, that following DNA damage acetylates histone H2AX (phosphorylated H2A) at Lysine 5, facilitating binding of the DNA repair protein NBS1 [26,27].

Histone “Readers” are non-histone proteins recruited to chromatin via domains (bromo, chromo and PHD) that recognize specific PTMs. Binding of “Readers” to chromatin can affect its structure or recruit additional factors e.g., transcription, DNA repair or replication. For example, H3K4me3 is recognized by bromodomain PHD finger transcription factor (BPTF), a component of the nucleosome remodeling factor (NURF) chromatin remodelling complex via a plant homeodomain (PHD) domain [58].

**Figure 2:**
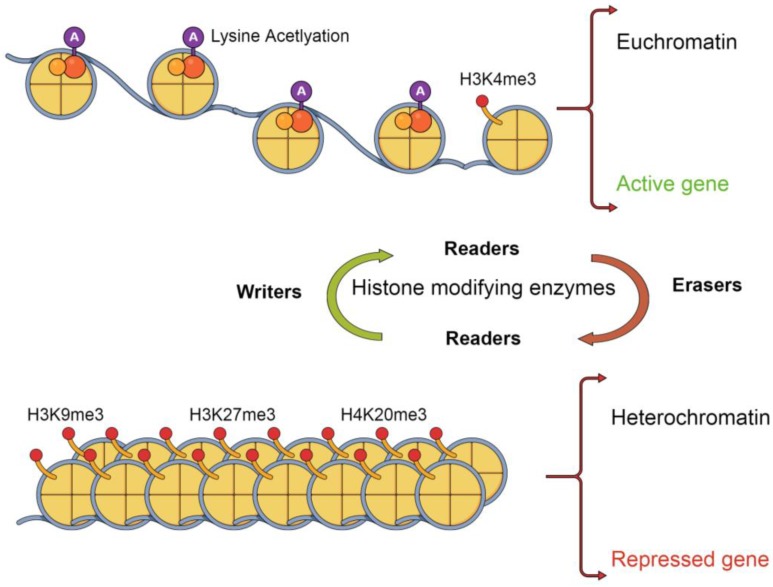
Posttranslational modifications of histones regulate gene expression. Effects of posttranslational modifications on gene regulation. Writers add posttranslational modifications, readers read the posttranslational modification landscape influencing further decisions and Erasers remove posttranslational modifications. TTS: transcription start site.

Histone “Erasers” remove marks left by the “Writers” altering chromatin accessibility and regulating many processes (Figure 2). For example, deacetylation can promote gene repression and silencing by removing the neutralizing acetyl charge from histones, leading to chromatin condensation [59]. Histone Lysine specific Demethylase 1 (LSD1) is an example of a specific “Eraser” that removes mono or di-methylated H3K4 and H3K9. LSD1 has been reported to be overexpressed in a number of tumours, and LSD1 inhibitors show potential as new targeted anti-cancer drugs [60].

As demonstrated by Writers and Erasers, histone PTM are dynamic and furthermore influence each other, engaging in “cross-talk” [61]. For example ubiquitination of H2B is required for H3K4 tri-methylation [62]. As these modifications can correlate with transcriptional activity, a “histone code” has been proposed, linking the PTM and chromatin conformation to downstream process and ultimately disease mechanisms, such as cancer [63]. Histone variants add another layer of complexity to the “histone code” as their incorporation influences and regulates specific cellular processes. Investigating the role histone variants (and their PTM) in cancer biology is an expanding and exciting area of research, to understand their mechanistic effects on basic molecular signalling and as potential biomarkers for disease.

## 4. Quantification of Nucleosomes PTMs as Tumour Biomarkers

The relationship between histone modifications and cancer has been the subject of much investigation in recent years, with Ac and Me, the most studied modifications. The loss of H4K16ac and H4K20me3 has been observed early in the tumorigenic process and occurs across a variety of cancer cell lines, in addition to global DNA hypomethylation [54]. Quantification of nucleosomes and specific PTMs show real potential as cancer biomarkers, both in tumours and importantly circulating in blood. We will first discuss global PTMs in tumour tissue as diagnostic or prognostic biomarkers.

### 4.1. Breast Cancer

Breast cancer is the most common cancer in women worldwide accounting for around 25% of cancers in women [64]. Breast cancer is a heterogenous disease with three established immunohistochemical biomarkers: Estrogen Receptor (ER), progesterone receptor (PR) and HER2 (human epidermal growth factor 2-receptor). Four molecular subtypes of breast cancer are defined by the presence or absence of these receptors (Luminal A (ER/PR positive), Luminal B (ER/PR, Her2 positive), HER2 (Her2 positive alone) and Basal-like (negative for all three receptors)) [65]. Each subtype displays different prognoses and has differing treatment strategies, based in their receptor status [66]. While these subtypes aid us in defining treatment and prognosis of breast cancer, further biomarkers are needed due to the heterogeneity observed between and within the subtypes [67]. New biomarkers enable us stratify patients more precisely, allowing optimization of therapy and monitoring response to treatment.

**Figure 3:**
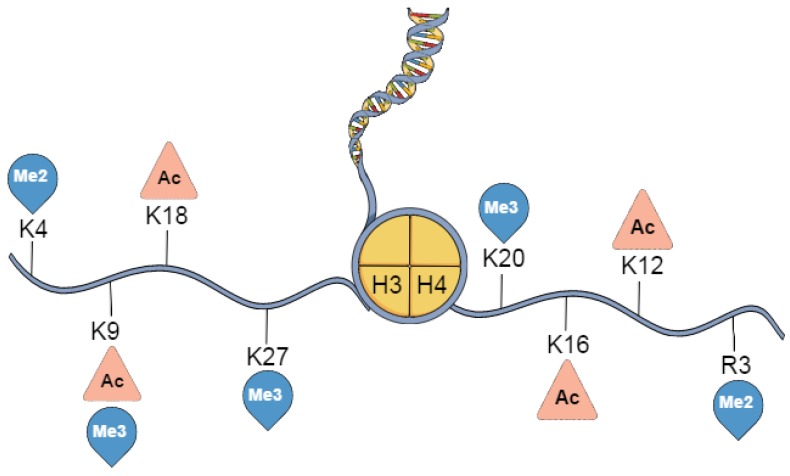
Posttranslational modifications to Histone 3 and 4 found in breast cancer. Key breast cancer associated modifications, to indicated residues, on Histone 3 (H3) or Histone 4 (H4). K: Lysine, R: Arginine, Ac: Acetylation, Me: Methylation, Di-Methylation (Me2), Tri-Methylation (Me3).

In breast cancer, a number of histone 3 and histone 4 PTMs have previously been examined (Figure 3). A study of 880 breast cancers correlated low expression of H3K9ac, H3K18ac, H4K12ac, H3K4me2, H4K20me2 and H4R3me2 with subtypes with a poorer prognostic outcome, such as, basal cancers [31]. Elevated global levels of acetylation and methylation correlated to better prognosis and were detected almost exclusively in luminal-like tumours. Finding that H4K16ac was low or absent in the majority breast tumours suggests H4K16ac may represent a biomarker for the early detection of breast cancer. Additionally, recent work indicated that global H4K12 hypoacetylation could be an early biomarker of ductal carcinoma in situ and invasive ductal carcinoma [68].

The repressive H4K20me3 has been associated with multiple cancers and loss of H4K20me3 in breast cancer tissue correlates with reduced survival and increased invasiveness. Supporting this, overexpression of the histone methyltransferases SUV420H1 and SUV420H2 restored H4K20me3 levels and repressed cancer-cell invasion in breast cancer cell lines [50]. Recently, genome-wide binding patterns of H3K4me3 (activating) and H3K27me3 (repressive) in normal mammary epithelial cells and three representative breast cancer subtype lines revealed subtype specific gene expression patterns and PTM-based gene classifiers that correlated significantly with relapse-free survival [69].

Histone Writers and Erasers have been linked to breast cancer—the histone acetyltransferase (HAT) hMOF (human males absent on the first), which acetylates H4K20, was significantly under-expressed in both primary breast cancer and medulloblastomas [70]. The Eraser LSD1 (Lysine-specific histone demethylase 1) is highly expressed in estrogen-receptor negative breast cancer and predicts aggressive biology [71] and inhibition of LSD1 resulted in growth inhibition of breast cancer cells. Recently, the use of HAT inhibitors as a potential new treatment for breast cancer has been explored [72].

### 4.2. Esophageal Cancer

Five year survival overall rates in the U.S. are 15%, 29%, and 65% for esophageal, gastric and colorectal cancer respectively [73]. Esophageal cancer tends to present late and has high recurrence rates following surgical intervention. There are currently no phase 5 cancer control study biomarkers available for oesophageal cancer. Low expression of H3K18ac and H3K27me3 has been shown to correlate to better prognosis in esophageal squamous cell carcinoma, particularly in early stages [42]. A subsequent study looking at high expression of H3K27me3 in patients who had received definitive chemoradiotherapy with curative intent showed a positive correlation with grade and tumour size [74]. The results suggested that H3K27me3 levels could stratify patients who with tumour stage 2 and 3 who had received chemoradiotherapy. In a recent study examining invasive esophageal squamous-cell carcinoma lines, high H3K79me2 expression correlated with increased invasive capacity and reduced survival.

### 4.3. Gastric Cancer

Gastric cancer is the leading cause of cancer-related mortality in developing countries with a low rate of diagnoses during early stages [75]. Carcinoembryonic antigen (CEA), Cancer Antigen 19-9 (CA19-9) and Cancer Antigen 72-4 (CA72-4) have all been proposed as potential biomarkers for gastric cancer. CA 72-4 has the highest sensitivity but none are ideal biomarkers for early detection and diagnosis, primarily used for detecting distant metastases or recurrence [76]. In gastric cancer, high expression of H3K9me3 significantly correlated with increased tissue and lymphovascular invasion and recurrence, resulting in worse prognosis [40]. Overexpression of H3K27me3 and the methyltransferase EZH2 (Enhancer of zeste homolog 2) have been shown to be independent prognostic factors for predicting survival in gastric cancer, and utilized together could potentially predict lymph node metastasis [77].

### 4.4. Colorectal Cancer

Colorectal cancer (CRC) is the second most frequent cause of death by cancer and there is a need for biomarkers to identify patients with stage 2 cancer (<20% chance of recurrence) who are candidates for adjuvant therapy [78]. CEA and micro-satellite instability (MSI) are potential biomarkers as prognostic factors. Mutations in the Kirsten Ras (KRAS) is associated with poorer survival in CRC oncogene [79] while around 10% of CRC patients have a B-Raf proto-oncogene serine/threonine kinase (BRAF) mutation [80]. DNA methylation plays a role in colorectal neoplasia and has also been investigated as a potential biomarker for CRC in predicting metastatic potential [81]. There are currently no established diagnostic biomarkers for CRC. H3K9e3 is increased in invasive colorectal cancer and correlated significantly with lymph node spread [82]. Interestingly, H3K9me3 is up-regulated in adeonomas and is a significantly increased in adenocarcinoma, suggesting H3K9me3 level is involved in the progression from adenoma to adenocarcinoma [83] Conversely, a study of H3K27me3 and its associated polycomb proteins EZH2, BMI1 (B lymphoma Mo-MLV insertion region 1 homolog) and SUZ12 revealed high expression of all four correlated with improved overall survival and recurrence-free survival [50]. Additionally, low nuclear expression of H3K4me3 and high expression of H3K9me3 and H4K20me3 in combination was found to be associated with better prognosis in early stage 1 and 2 CRC [50]. More recently, combinations of modifications have been investigated in early stage colon cancer, low nuclear expression of H3K4me3 and high expression of H3K9me3 and H3K4me3 correlated to increased survival and longer local and distant recurrence free-survival [56]. It has also been demonstrated that PTMs can exhibit an age-dependent prognostic value in colorectal cancer [84], where increased H3K27me3 and H3K9ac was found in older patients with poor survival or outcomes (death/recurrence), compared with decreased expression in the no-event group.

## 5. Histone Variants

Histone variants (H2A.Z, macroH2A, H2A.B, H3.3) increase chromatin complexity and when dysregulated can contribute to carcinogenesis [85,86]. Recent studies have examined the potential role of histone variants as biomarkers in cancer, in particular variants of H2A and H3.

In breast cancer, the H2A.Z variant is induced by estrogen via the activation of MYC [87]. High-intensity staining of H2AZ correlates with increased lymph node metastases and poorer overall survival and when combined with ER/PR or Her2 staining, increases their prognostic power.

The H2A variant macroH2A (mH2A) is a repressive histone approximately three times the size of the canonical H2A which contributes to X-chromosome silencing, as well as being associated with condensed chromatin and inactive alleles of imprinted genes [88]. In anal carcinoma, expression of mH2A was lost in 38% of high-grade anal neoplasia and 72% anal squamous cell carcinoma. Loss of mH2A also correlated with recurrence and Human Papilloma Virus (HPV) negative tumours, suggesting the potential of mH2A as a biomarker in the progression of anal carcinoma [89].

Following DNA damage, a key signalling event initiating the DNA damage response pathway is the phosphorylation of histone variant H2A.X at serine 139 (now termed γ-H2A.X). Endogenous foci of this variant are rare in normal tissue, however in breast cancer high levels of γ-H2A.X foci was associated with triple negative subtype and a worse overall prognosis [90] and in colorectal tissue is associated with more malignant cancer behavior and poor patient survival [91].

The detection and quantification of both histones and their associated PTM has shown great promise as tumour biomarkers. However, this requires an invasive biopsy of an established tumour-reducing their utility. To fully leverage these biomarkers, deploying them as early prospective markers using non-invasive sampling needs to be explored and established.

## 6. Circulating Nucleosomes as Cancer Biomarkers

It has been shown that nucleosomes are released into plasma following cell death and apoptosis [92] and many are then captured by macrophages [93]. These are carried as predominantly oligonucleosomes or mononuclesomes with circulating cell-free DNA (cfDNA). [94] cfDNA and circulating tumour DNA (ctDNA) comprises a small proportion of total circulating DNA and is an attractive potential biomarker for guiding patient treatment and informing prognosis, and have been extensively examined as a part of a potential “liquid biopsy” [95]. ctDNA has proven challenging to quantify due its low numbers in circulation and the difficulty in discriminating ctDNA from cfDNA. New techniques such as Polymerase Chain Reaction (PCR) [96], beads, emulsion, amplification and magnetics (BEAMing) [97] and next-generation sequencing (NGS) [98] has enabled the identification of ctDNA as potential liquid biopsies. ctDNA, in combination with circulating tumour cells, are attractive biomarkers because of their non-invasive accessibility and have been examined as potential liquid biopsies in breast, colorectal, pancreatic, haemotological, lung and urological malignancies [99,100,101,102,103,104].

Nucleosomes are stable structures in circulation with an ≈7% decrease in nucleosome concentration in each collected sample, per year in storage [105] and can be detected using enzyme linked immunosorbent assays (ELISA) in serum and plasma. A recent study sequenced cfDNA from plasma and found that the cell architecture, structure and expression of the cfDNA nucleosome correlates with the cell of origin [106]. Nucleosomes are detected at increased rates in cancer due to higher cellular turnover [107], cell death due to cytotoxic chemotherapeutic treatments ([108]) and in non-neoplastic disease processes (such as stroke, sepsis and trauma) when the mechanism of their removal has been overloaded. A number of studies have shown an elevated level of circulating nucleosomes in cancers including lung, breast, colorectal and prostate cancer in comparison to healthy controls, particularly in cases of advanced cancer [109,110]. However, when compared to levels in patients with many benign diseases, the difference was not statically significant, reducing their clinical utility for detecting cancer.

As circulating nucleosomes are released in response to chemotherapeutic treatments, studies have investigated monitoring changes in circulating nucleosomes levels to observe tumour responses. Quantification of circulating nucleosomes has been investigated as a method to identify patients not responding to therapy, before or early in the treatment regime, allowing modification of treatment regimes, ultimately sparing patients from the systemic side-effects of ineffective chemotherapy [111]. In patients with advanced lung cancer, pre-therapeutic levels of circulating nucleosomes were significantly lower in patients who responded to chemotherapy (and significantly achieved remission) and these patients exhibited a greater decrease in circulating nucleosomes following the 1st cycle of the chemotherapy regime [112].

In a study examining potential biomarkers for predicting response to neoadjuvant chemotherapy in breast cancer, they found higher pre-therapeutic levels of nucleosomes in patients who did not respond to neo-adjuvant chemotherapy, compared to those who did.

A recent trial described modelling in patients with colorectal cancer and liver metastases where combining circulating nucleosomes levels (24 h post radiation treatment) with pre-therapeutic levels of CRP and AST improved the prognostic model (compared to CRP and AST alone) [113].

## 7. Quantifying PTM of Circulating Nucleosomes as Biomarkers

Recent studies identified post-translational modifications on circulating nucleosomes in cancer patients and explored their potential as biomarkers. H3K9me3 and H4K20me3 are involved in the formation of heterochromatin (Table 3, Figure 1) and have been linked to breast and colorectal cancer [114]. H3K9me3 and H4K20me3 levels were significantly reduced in the plasma of colorectal cancer patients, compared to healthy controls [33]. H3K27me3 has been associated with breast, ovarian and pancreatic cancer and also with poorer prognosis [52]. Supporting previous results, reduced levels of H3K27me3 and H4K20me3 were identified in colorectal cancer patients, in comparison to healthy controls. However, unlike previous reports the levels of H3K9me3 were similar between the two groups. Encouragingly, when H3K27me3 and H4K20me3 were combined the results showed greater area under the curve (0.769), and sensitivity of 49.2% at 90% specificity for colorectal cancer [52].

In pancreatic cancer, where 5-year overall survival remains at 6%, CA19-9 (Carbohydrate antigen 19-9) is the current gold standard biomarker, but is only recommended for monitoring response to treatment [115]. However, a recent Swedish study investigated a panel of five epigenetic biomarkers in circulating nucleosomes—5MC (nucleosome-associated methylated DNA), H2A.Z, H2A.A, H3K4me2 and H2AK119Ub in patients with resectable pancreatic cancer and found that they were superior to CA19-9 [116] (compared to benign pancreatic disease and healthy controls). Combining CA19-9 with four of these epigenetic biomarkers gave a sensitivity of 92% at 90% specificity in diagnosing pancreatic cancer.

More recently the effects of pre-analytical variables were investigated to determine the durability of circulating nucleosomes. Stasis, contamination with white cells, within-day variation, varying time before centrifugation, colonoscopy and surgical trauma had no significant influence on the level of 5-methylcytosine DNA (5mC) or H3K9me3 in circulating nucleosomes [117]. The 5mC and H3K9me3 levels were significantly lower in cancer patients compared to healthy individuals [117].

Recently circulating microRNAs have shown great promise as biomarkers of cancer [118,119,120,121]. Combining circulating microRNA with nucleosome quantification and nucleosome PTMs may led to a more specific and sensitive bio-signature, and in themselves may influence nucleosome levels or PTM by regulating key proteins.

## 8. MicroRNA Regulation of Histone PTM

MicroRNAs (miRNAs or miR) are 20–25 base pair non-protein coding RNA that operates as gene regulators by inhibiting degradation of their target messenger RNA. Currently, >2000 miRNAs have been identified in humans and are important regulators of gene expression [122]. Importantly, aberrant expression of specific miRNA contributes to the initiation and expression of cancer [123]. miRNAs can operate as oncogenes and tumour suppressor genes and their expression can be altered during the process of carcinogenesis. They are detectable in circulation and show great potential as biomarkers in cancer [118,119,120].

It has been shown that post-translational modification of histones can affect the expression of miRNAs. miR-125-b1 contributes to carcinogenesis by regulating genes such as *BAK1*, an anti-apoptotic gene [124] and its down-regulation is associated with a poor prognosis in breast cancer. At the miR-125b-1 promoter, H3K27me3 and H3K9me3 were enriched in Luminal A and triple-negative breast cancer cell lines respectively and in the Luminal A cells, H3K27me3 was responsible for miR-125b-1 repression [125].

## 9. Conclusions

The measurement of absolute levels of circulating histone and quantification of PTM in these circulating histones provide an exciting new avenue for the non-invasive diagnosis and monitoring of cancer progression and treatment. Further work investigating links between circulating nucleosome PTM and circulating miRNA will not only provide clinically relevant biomarkers, but reveal information related to the fundamental mechanisms underpinning cancer progression response to treatments. Clinically utilizing the real-time (or near real-time) quantification or monitoring of circulating nucleosomes or circulating nucleosomes posttranslational modifications will provide a potentially quick, cheap and robust method for detecting cancer and monitoring the response of cancers to treatments.

## Figures and Tables

**Table 1:** Histone 2B: post-translational modifications (PTM) quantification as cancer biomarkers.

Modification	Writer	Eraser	Function	Cancer role	Reference
Histone 2B					
Global hypoacetylation & hypomethylation	P300, ATF2		Transcriptional activation	Low level-Prostate	[28]

**Table 2:** Histone 3: PTM quantification as cancer biomarkers.

Modification	Writer	Eraser	Function	Cancer Role	Reference
Histone 3					
H3K4Ac		HDAC 3	Transcriptional activation	Low level—poorer prognosis in oral squamous cell carcinoma	[29]
H3K4Me	SETD 7	KDM 1A	Transcriptional activation	High levels in locally confined prostate cancer	[30]
H3K4Me2	NSD 3	KDM 1A	Transcriptional activation	Low levels in breast, pancreatic, renal and lung carcinomas of worse prognosis Low levels—higher rate of prostate cancer recurrence	[31,32,33]
		KDM 5A			
		KDM 5D			
H3K4Me3		KDM 2B	Transcriptional activation Transcriptional elongation	High expression associated with poorer prognosis in hepatocellular carcinoma	[34]
	MLL	KDM			
	MLL 3	KDM 5A			
	MLL 4	KDM 5B			
	PRDM 9	KDM 5C			
	SETD 1A	KDM 5D			
	SETD 1B	PHF 8			
		NO 66			
H3K9Ac	Gcn 5	SIRT 1	Transcriptional activation Telomere metabolism and function	Low level—breast cancer of poorer prognostic subtype Low levels—poorer prognosis in non-small cell lung cancer	[31,35]
		SIRT 6			
H3K9Me	G9a	KDM 1A	Transcriptional repression	Decreased levels indicates poor prognosis in renal cell cancer Decreased levels in bladder cancer	[36,37]
		KDM 3 A & B			
H3K9Me2		PHF 8	Transcriptional repression and initiation	Low levels in prostate & pancreatic cancer Low levels—poorer outcome in prostate and renal cancer High levels in bladder cancer, correlates with pT stage and grade	[30,32,33,37]
		KMD 1A			
	Glp 1	KDM 1B			
	G9a	KDM 3A			
	PRDM 2	KDM 3B			
		KDM 4C			
		KDM 4D			
		KDM 7			
H3K9Me3	SETDB 1	KDM 4A	Transcriptional repression and initiation Indexing of pericentromeric chromatin	Low levels in circulating nucleosomes in colorectal cancer, high in breast cancer High levels correlates with poorer survival and increased recurrence in gastric cancer Predicts survival in acute myeloid leukemia High levels in bladder cancer, correlates with pT stage and grade	[37,38,39,40,41]
	SETDB 2	KDM 4B			
	SUV39H 1 & 2	KDM 4C			
		KDM 4D			
H3K18Ac	P300		Transcriptional activation	Low levels—breast cancer of poorer prognostic subtype Low levels—better prognosis in esophageal squamous cell carcinoma, especially early Lower levels in muscle-invasive bladder cancer compared to non-muscle invasive and normal tissue Correlates to higher tumor grade in prostate cancer Low levels—poorer survival in pancreatic cancer	[31,32,42,43,44]
	CBP				
	Elp3				
H3K27Ac	P300		Transcriptional activation	Up-regulated in colorectal cancer	[45]
	CBP				
H3K27Me	EZH 1		Transcriptional activation	Low levels correlate to decreased survival in renal cell carcinoma (in addition to H3K27me2 &H3K27me3)	[46]
	EZH 2				
	Glp 1				
	G9a				
H3K27Me2	EZH 1	KDM 6B	Transcriptional repression	Lower levels correlate with poorer survival in colorectal cancer with liver metastases	[47]
	EZH 2	KDM 7			
	NSD 3	PHF 8			
H3K27Me3	EZH 2	KDM 6A	Transcriptional repression	Low levels—poorer prognosis in breast, ovarian and pancreatic cancer High expression correlates with vascular invasion and poorer prognosis in hepatocellular cancer High expression—increased survival in colorectal cancer High expression—better prognosis in non-small cell lung cancer High expression—poorer prognosis in oral squamous cell carcinoma	[48,49,50,51,52]
	NSD 3	KDM 6B			
H3K36Me2	SETMAR	KDM 2A	Double strand repair & Non-homologous end joining	High level—correlates to histological subtype in primary colorectal cancer	[47]
	NSD 1	KDM 2B			
	SMYD 2	KDM 8			
	ASH 1L				
H3K36Me3	SET D2	KDM 4A	Antagonises PRC2-mediated H3K27 methylation	High level—correlates to lymph node spread in primary colorectal cancer	[47]
	NSD 2	NO66			
H3K56Ac		HDAC 1	DNA double stranded break repair	High expression—poorer prognosis in colorectal cancer	[53]
	CBP	SIRT 1			
	P300	SIRT 3			
		SIRT 6			

**Table 3:** Histone 4: PTM quantification as cancer biomarkers.

Modification	Writer	Eraser	Function	Cancer Role	Reference
Histone 4					
H4Rme2	PRMT 1	JMJD 6	Facilitates acetylation of H3 and H4 resulting in gene activation	Low levels in breast cancer of poorer prognosis	[31]
	PRMT 5				
	PRMT 6				
H4K12Ac	Gcn 5		Histone deposition, telomere silencing Transcriptional activation, DNA repair Transcriptional activation	Low levels in breast cancer of poorer prognosis	[31]
	KAT1				
	Tip60				
	P300				
	CBP				
	MYST2				
H4K16Ac	Gcn 5		DNA damage response & double-strand repair	Loss—Hallmark of cancer Low/absent in majority of breast cancers	[31,54]
	P300	SIRT 1			
	CBP	SIRT 2			
	hMOF				
H4K20Me3	SUV420H1		Gene silencing Indexing of pericentromeric chromatin	Loss—Hallmark of cancer Reduced levels – independently associated with lower disease-free survival in breast cancer High expression (in combination with high H3K9Me3 & low H3K4Me3) correlates to better prognosis in colorectal cancer Significantly lower levels in circulating nucleosomes of colorectal cancer patients	[54,55,56,57]
	SUV420H2				
	NSD2				
